# Changes and predictive value for treatment outcome of the compulsive exercise test (CET) during a family-based intervention for adolescents eating disorders

**DOI:** 10.1186/s40359-018-0265-9

**Published:** 2018-11-15

**Authors:** Ingemar Swenne

**Affiliations:** Department of Women’s and Children’s Health, Uppsala University, S-75185 Uppsala, Sweden

**Keywords:** Eating disorder, Adolescent, Compulsive exercise, Family-based treatment

## Abstract

**Background:**

The aim of this study was to explore changes in the Compulsive Exercise Test (CET) following a family-based intervention in adolescents with restrictive eating disorders (ED). It was hypothesized that compulsive exercise would improve with successful intervention against the ED but also that a high level of compulsive exercise at presentation would be associated with a less favourable outcome.

**Method:**

The CET, the Eating Disorders Examination-Questionnaire (EDE-Q), and body mass index were available for 170 adolescents at presentation and at a one-year follow-up. Treatment was a family-based intervention and included that all exercise was stopped at start of treatment. Recovery was defined as EDE-Q score < 2.0 or absence of an ED at an interview.

**Results:**

Exercise for weight control and for avoiding low mood, which are related to ED cognitions, decreased in recovered patients. Exercise for improving mood did not change in either recovered or not recovered patients. The CET subscale scores at presentation did not independently predict recovery.

**Conclusion:**

Compulsive exercise is one of several ED related behaviours which needs to be targeted at the start of treatment. With successful treatment it decreases in parallel with other ED related cognitions and behaviours but without a loss of the ability to enjoy exercise.

## Background

Anorexia nervosa (AN) and other restrictive eating disorders (ED) are severe psychiatric disorders, commonly presenting in adolescent females and characterised by a restriction of food intake causing weight loss and a distorted body perception. Compulsive and/or excessive exercise is a common feature of adolescent ED although not a prerequisite for the diagnosis [1–3]. In early studies, the role of exercise was seldom included in studies of treatment outcome of AN [4]. More recently it has been established that compulsive exercise is associated with strong ED cognitions [1–3, 5–7] and that it influences treatment outcome negatively [8, 9]. The definition of compulsive exercise in ED has varied but it is generally agreed that the distinguishing feature is not the amount or intensity of exercise but the qualitative dimension of compulsivity [6]. The concept of compulsive exercise has been further developed since exercise is driven not only by a desire to control weight and shape but also by its effects on mood [6, 10]. If exercise is prevented there is an increase of anxiety and negative affect which would be reduced by resuming exercise. Exercise could also be performed for the experience of positive affect. When not being able to exercise, this agreeable experience would be missed [6]. Therefore, a multidimensional construct is necessary to describe exercise in ED [6, 11].

The compulsive exercise test (CET) [11, 12] taps these different aspects of exercise. The CET subscales for weight control exercise and for avoidance of negative mood are strongly correlated with ED cognitions in non-clinical samples [10, 12, 13] and in adolescents with ED [14, 15]. On the other hand, exercise for a positive experience is only weakly, if at all, related to ED cognitions in either type of sample [10, 12–15]. So far little is known of how CET scores change during treatment of an ED. Considering that the different cognitive aspects of exercise are not all correlated with ED cognitions it is conceivable that an intervention against the ED affects them differently.

In the present study, the changes of CET scores following a family-based intervention for adolescents with restrictive ED have been investigated. The intervention was directed against ED cognitions and behaviours which included stopping all exercise at the start of treatment. In view of the fact that compulsive exercise and ED cognitions are closely related it was hypothesized that exercise both for weight control and for avoiding negative mood would improve following successful intervention. It was furthermore hypothesised that high levels of these aspects of exercise at presentation would be associated with a less favourable outcome of the ED.

## Methods

### Participants

The Eating Disorders Unit (EDU) at The Department of Child and Adolescent Psychiatry of the Uppsala University Hospital is the only specialised ED unit in the county. It provides treatment to all patients with ED and < 18 years of age in the county (population 345,481 of which 70,424 < 18 years on Dec 31 2013) [16]. During the period March 2012 – June 2016 297 new patients were assessed and diagnosed with a restrictive ED. Two hundred and seventy-seven started treatment at the EDU. One year after presentation (12,4 ± 0,8 months, range 10–15) 198 (71%) of these attended a follow-up interview. Complete data including growth charts with premorbid weight, weight at presentation and at follow-up, and all the self-report instruments were available for 170 (61%). This a secondary analysis of the data since the sample partly overlaps with that of a previous analysis of predictors of outcome in our treatment programme [17].

### Procedure

Assessment of new patients was performed by a paediatrician with experience of ED. An interview with the adolescent and at least one parent included the history of the ED, and a general medical history to assess somatic and psychiatric comorbidity. Weight and height were measured in underwear only, and a physical examination performed. Blood samples were obtained to exclude hitherto unknown comorbid disease and to evaluate the impact of weight loss on metabolism and nutritional state. Growth charts were procured from the school health services for objective measures of premorbid growth and weight changes. An ED diagnosis was established, and treatment immediately started (see below). A second appointment was scheduled 1 week later. At this meeting assessment was reviewed and instruments administered. Measurements of weight and length were registered at 1 week, 1 month and 3 months after start of treatment. One year after start of treatment a face-to-face follow-up interview was performed, usually by the therapist who had seen the patient/family for the past year. This was to map ED ideation and ED behaviours such as restricting food, vomiting or exercising for weight control and determine whether the adolescents fulfilled criteria for an ED. The follow-up visits included measurement of weight and length and administration of the self-report questionnaires used at presentation. The procedure for assessment, start of treatment and follow-up has been described in detail [17, 18].

The protocol was approved by the Ethics Committee of the Faculty of Medicine of Uppsala University.

### Study measures

ED diagnoses were according to the Diagnostic and Statistical Manual of Mental Disorders, Fifth edition (DSM-5). The earliest part of the sample had been diagnosed according to DSM-IV and was retrospectively recoded into DSM-5 criteria. Body mass index (BMI) was calculated as weight/height^2^ (kg/m^2^) and recalculated into BMI standard deviation scores (BMI SDS), which constitutes a measure of leanness corrected for age and height [19]. BMI SDS below − 2.00 was used as the weight criterion for anorexia nervosa (AN) [16, 20]. At presentation weight loss was calculated as the difference between weight at presentation and the highest recorded premorbid weight. At follow-up weight suppression was calculated as the difference between BMI SDS at follow-up and BMI SDS at the highest premorbid weight.

A recently validated Swedish version [14] of the CET [12] was used. The CET is comprised of five subscales with altogether 24 items which assess cognitive and behavioural aspects of compulsive exercise. Responses are scored from zero to five and averaged for each subscale with high scores representing a high degree of compulsive exercise. The subscale “avoidance and rule-driven behaviour” (e.g., “If I cannot exercise I feel low and depressed”) taps regulation of low mood by exercise. “Weight control exercise” (e.g., “I exercise to burn calories and lose weight”) is related to modification of weight and shape by exercise. “Mood improvement” (e.g., “Exercise improves my mood”) is associated with enhancing good mood. “Lack of exercise enjoyment” (e.g., “I do not enjoy exercising”) and “exercise rigidity” (e.g., “I follow a set routine for my exercise sessions”) is related to obsessional and rigid aspects of exercise. To the CET was added a question on exercise frequency: “How many days per week do you usually exercise?”

The Eating Disorders Examination-Questionnaire youth version (EDE-Q) [21] was used to assess ED ideation. Twenty-three items are subdivided in the four subscales “eating restraint”, “eating concern”, “weight concern” and “shape concern”. Items are scored from zero to six and averaged for each subscale with high scores representing a high degree of ED ideation. A global score is calculated by averaging the subscale scores. The Montgomery-Åsberg Depression Rating Scale-Self report (MADRS-S) [22] was used to assess depressive symptoms. Nine items are scored from zero to six and summed with high scores representing high depressive symptomatology.

Recovery was defined by two separate measures: 1) EDE-Q global score < 2.0. This cut-off corresponds to the mean + 1 SD of the score of adolescent reference samples [21, 23] and to the clinically significant score in a Swedish sample [24]. 2) Not meeting diagnostic criteria for an ED at the interview at the one-year follow-up.

### Treatment

Treatment is family based and underscores the role of the parents in the care of their child. In Sweden this is supported by the social security system which allows reimbursed parental leave to care for a sick child under the age of 18. Treatment is an outpatient intervention, which can be intensified by adding day treatment [16]. In-patient treatment is not part of the treatment programme and used only in emergency situations [25].

The first step of the treatment programme has an aim of stopping on-going weight loss and bringing meal routines back into order. This is underscored already at presentation [18]. Parents get advice on their role in the re-establishment of their family meal practices. They are advised as to what is a normal-size meal and to implement normal table manners. Routines for avoiding vomiting after meals are suggested. Attending school is advised against as long as meal pattern and normal eating have not been re-established. All forms of exercise are stopped at the start of treatment.

The second step of the programme follows when meal routines have been re-established although support at all meals is necessary. The aim is now to restore weight by 0.5–1 kg/week. A final step starts with a gradual reintroduction into school. This requires that eating has been normalised and that weight deficit has decreased considerably. Vigilance over daily routines can be reduced although meal support may still be needed. Exercise is reintroduced, usually what the adolescent took part in before falling ill, provided that it can be done safely without recurrence of ED cognitions. The possibility of co-morbid psychiatric disease may now be reassessed and treatment of problems outside the core features of the ED introduced. For example, low self-esteem, over-evaluation of weight and shape, perfectionism and/or interpersonal difficulties can be addressed to prevent relapse. The programme does not have a fixed number of sessions, but the steps are goal oriented. Duration of treatment varies with a median of ten sessions (range 4–36) over a median of 9 months (range 3–24). At the one-year follow-up approximately 50% of the patients have finished treatment, 35% are still in treatment at the EDU and 15% have been referred to other psychiatric services or has discontinued treatment against advice. At follow-up patients have, with few exceptions, been reintroduced into exercise. The treatment programme has previously been described in detail [16–18].

The treatment programme is strongly influenced by FBT [26]. It differs in that parents are suggested interventions at the first session rather than empowering them to find their own solutions to re-establish meal routines. It also differs in that cognitive behavioural therapy is used for comorbid disorders and remaining ED-related issues. The important similarity with FBT is the emphasis on that it is the parents who should take a leading role against the ED and re-establish family routines.

### Data analysis

Statistical analyses were performed in SPSS 20.0.0. Values are given as means ± SD. Differences in weight and psychometrics measures were compared using Student’s t-test for independent samples for continuous data and Chi-square tests for categorical data. To minimize the risk for mass significance and type 1 error the significance level was set at *p* < 0.01. To analyse predictors of outcome logistic regression analyses were used. In these analyses either one of the outcome measures “EDE-Q global score <2.0” or “no ED at the follow-up interview” was entered as the dependent variable. In a first set of analyses each of the different CET subscales was entered as an independent variable to determine whether any one was related to outcome prior to correction for the other predictors. In a second set of analyses BMI SDS at presentation, EDE-Q global score at presentation, weight gain at 3 months and weight suppression at follow-up were entered together as independent variables since they have previously been shown to predict outcome [17]. The individual CET subscales were then forced into the models to asses if they independently added to and predicted outcome.

## Results

### Patient characteristics

Characteristics at presentation for the 170 patients are given in Table 1. Twenty-six (15%) patients had restrictive AN. The remainder had a restrictive eating disorder with features of AN but not reaching the weight criterion (other specified feeding and eating disorders-restrictive subtype; OSFEDr). In these there was a wide variation of BMI SDS and of weight loss at presentation. Reporting exercise for weight control was common but only a fifth of the adolescents vomited for weight control. Twenty-eight (17%) were diagnosed with depression.

**Table 1:** Characteristics at assessment of 170 adolescents presenting with a restrictive eating disorder

Gender (M/F)	10/160
Age (years)	15,0 ± 1,6 (range 11.4–17.8)
BMI SDS	−0,84 ± 1,19 (range − 3.69 – 2.47)
Weight loss (kg)	6,4 ± 5,4 (range 0–27.4)
Duration (months)	9,0 ± 7,4 (range 2–48)
Reporting vomiting	29 (17%)
Reporting exercise	137 (81%)
Reporting self-destructive behaviour	23 (14%)
ED diagnoses (AN/OSFEDr)	26/170 (15/85%)
Depression	28 (17%)
Reported exercise frequency (days/week)	3,7 ± 1,9
CET total score	2,66 ± 0,87
CET avoidance and rule driven behaviour	2,60 ± 1,40
CET weight control	3,05 ± 1,35
CET mood regulation	3,59 ± 1,20
CET lack of exercise enjoyment	1,37 ± 1,08
CET exercise rigidity	1,47 ± 1,35
EDE-Q global score	2,9 ± 1,6
MADRS-S	17 ± 10

Values are means ± SD

*AN* anorexia nervosa, *BMI* body mass index, *CET* compulsive exercise test, *ED* eating disorder, *EDE-Q* eating disorders examination-questionnaire, *MADRS-S* Montgomery-Åsberg depression rating scale – self report, *OSFEDr* other specified feeding and eating disorders – restrictive subtype, *SDS* standard deviation score

Chronic somatic diseases were present in 20 (12%) patients; of these five had type 1 diabetes, five had coeliac disease and two had hypothyreosis. Psychiatric comorbidity, other than depression, and in all but one neuropsychiatric diseases, was present in 12 (7%). None of the comorbid diagnoses was judged to influence the ability to exercise.

### Patient characteristics in relation to outcome

When recovery was defined as an EDE-Q score < 2.0, 125 (74%) patients met the criterion (Table 2). At presentation, these patients differed from those not recovered by lower EDE-Q global scores and lower MADRS-S scores. At presentation, the recovered patients had lower scores on the “avoidance and rule driven behaviour”, “weight control exercise” and “exercise rigidity” subscales of the CET but did not report different exercise frequencies. Weight gain during the first 3 months of treatment was greater for recovered patients.

**Table 2:** Characteristics of adolescents in a family-based intervention of 170 adolescents with restrictive eating disorders

	EDE-Q global score at 1-year follow-up		ED at 1-year follow-up	
	<2.0	≥2.0	No ED	Persisting ED
n	125	45	99	71
At presentation				
Gender (M/F)	9/116	1/44	8/91	2/69
Age (years)	14.9 ± 1.6	15.3 ± 1.4	15.1 ± 1.6	15.0 ± 1.5
BMI SDS	−0.98 ± 1.21	− 0.45 ± 1.05	− 0.80 ± 1.23	−0.90 ± 1.14
Weight loss (kg)	6.2 ± 5.3	7.0 ± 5.6	6.2 ± 5.6	6.7 ± 5.1
Duration (months)	8.6 ± 7.1	9.9 ± 8.4	9.8 ± 8.0	7.8 ± 6.6
Reporting vomiting	20 (16%)	9 (20%)	18 (18%)	11 (15%)
Reporting exercise	99 (79%)	38 (84%)	80 (80%)	57 (80%)
Depression	17 (14%)	11 (24%)	14 (14%)	14 (20%)
Reported exercise frequency (days/week)	3.7 ± 2.0	3.6 ± 1.8	3.7 ± 1.9	3.2 ± 1.6
CET avoidance and rule driven behaviour	2.4 ± 1.4	3.1 ± 1.2^**^	2.5 ± 1.4	2.7 ± 1.4
CET weight control	2.8 ± 1.4	3.7 ± 1.0^***^	3.0 ± 1.3	3.2 ± 1.4
CET mood regulation	3.5 ± 1.3	3.7 ± 1.0	3.5 ± 1.1	3.6 ± 1.3
CET lack of exercise enjoyment	1.4 ± 1.1	1.4 ± 1.1	1.4 ± 1.0	1.3 ± 1.1
CET exercise rigidity	2.6 ± 1.3	3.1 ± 1.1^**^	2.6 ± 1.2	2.8 ± 1.2
EDE-Q global score	2.6 ± 1.7	3.7 ± 1.3^***^	2.8 ± 1.7	3.2 ± 1.6
MADRS-S	16 ± 10	21 ± 9^**^	16 ± 10	19 ± 10
At 1-year follow-up				
BMISDS	0.03 ± 0.89	0.01 ± 1.05	0.13 ± 0.89	−0.12 ± 0.97
Weight gain at 3 months (kg)	4.6 ± 3.1	2.1 ± 4.0^***^	4.7 ± 3.1	3.0 ± 3,7^**^
Weight gain at 1 year (kg)	8.2 ± 4.58	4.7 ± 7.7^***^	8.0 ± 4.7	6.3 ± 6.8^***^
Weight suppression (SDS)	0.35 ± 0.81	0.91 ± 0.95^***^	0.37 ± 0.81	0.68 ± 0.96
Depression	9 (7%)	15 (33%)^***^	6 (6%)	18 (25%)^**^
ED diagnoses (noED/AN/BN/OSFEDr)	96/1/1/27 (77/< 1/< 1/22%)	3/3/3/36^***^ (7/7/7/79%)	0/0/0/0	0/4/4/63^***^ (0/6/6/88%)
Reported exercise frequency (days/week)	2.8 ± 1.5	3.1 ± 1.7	2.8 ± 1.4	2.9 ± 1.8
CET avoidance and rule driven behavior	0.9 ± 0.9	3.1 ± 1.1^***^	0.9 ± 0.9	2.4 ± 1.4^***^
CET weight control	1.1 ± 0.8	3.5 ± 1.2^***^	1.1 ± 0.9	2.6 ± 1.5^***^
CET mood regulation	3.1 ± 3.4	3.7 ± 1.0^**^	3.2 ± 1.4	3.5 ± 1.2
CET lack of exercise enjoyment	1.1 ± 1.2	1.4 ± 0.9	1.0 ± 1.1	1.4 ± 1.1
CET exercise rigidity	1.8 ± 1.2	2.9 ± 1.2^***^	1.8 ± 1.2	2.5 ± 1.3^**^
EDE-Q global score	0.7 ± 0.6	3.5 ± 0.9^***^	0.6 ± 0.6	2.5 ± 1.5^***^
MADRS-S	7 ± 6	20 ± 10^***^	7 ± 6	16 ± 11^***^

*AN* anorexia nervosa, *BMI* body mass index, *BN* (subthreshold) bulimia nervosa, *CET* compulsive exercise test, *ED* eating disorder, *EDE-Q* eating disorders examination-questionnaire, *MADRS-S* Montgomery-Åsberg depression rating scale – self report, *OSFEDr* other specified feeding and eating disorders – restrictive subtype, *SDS* standard deviation score

Values are means ± SD. Significance of difference between recovered adolescents (EDE-Q < 2.0 or absence of an ED) and those with a persisting disease: ^**^ *p* < 0.01, ^***^p < 0.001 by Student’s t-test for continuous data and Chi-square test for categorical data

At the one-year follow-up recovered patients had greater weight gain, less weight suppression, and a lower prevalence of depression. On the CET they had, in comparison with non-recovered, considerably lower scores on the “avoidance and rule driven behaviour”, “weight control exercise” and “exercise rigidity” subscales while “mood regulation” was only little lower. Patients who were not recovered had CET scores at the level observed at presentation.

Of the patients meeting the EDE-Q criterion for recovery twenty-six (21%) were not considered recovered at the one-year follow-up interview. Ninety-nine (58%) patients did not fulfil diagnostic criteria for an ED at the follow-up (Table 2). At presentation, they did not differ from those not recovered. Weight gain during the first 3 months of treatment was greater for recovered patients. At the follow-up they had greater weight gain and a lower prevalence of depression. On the CET recovered patients, in comparison with non-recovered, had considerably lower scores on the “avoidance and rule driven behaviour” and “weight control exercise” subscales while “exercise rigidity” was only little lower and “mood regulation” and “lack of exercise enjoyment” did not differ. Patients who were not recovered had CET scores at the level observed at presentation.

### Predictors of outcome

When the CET subscales were entered as independent variables in a logistic regression analysis without correction for other predictors “avoidance and rule driven behaviour” and “weight control exercise” predicted the outcome “EDE-Q score <2.0” (Table 3). The subscale “mood regulation” did not predict outcome. The outcome “no ED” was not predicted by any of the CET subscales. Outcome measures were subsequently analysed against one-unit changes of BMI SDS at presentation, EDE-Q global score at presentation, weight suppression at follow-up and a 3-kg weight gain at 3 months. Such analyses replicated the associations with favourable outcome at the one-year follow-up previously observed [17] (Table 4). Thus, the outcome “EDE-Q score < 2.0” was associated with a lower EDE-Q global score at presentation, a higher 3-month weight gain at the start of treatment and a lower weight suppression at the 1-year follow-up. The outcome “not having an ED” was associated with higher BMI SDS at presentation, a lower EDE-Q global score at presentation and a higher 3-month weight gain at start of treatment. When either “CET weight control exercise”, “CET avoidance and rule driven behaviours” or “CET mood regulation” scores at presentation was forced into the models prediction of outcome was not improved.

**Table 3:** Prediction of one-year outcome of family-based treatment of 170 adolescents with restrictive eating disorders

Outcome	Predictor	Odds ratio	95% CI	*p*
EDE-Q global score < 2.0	CET avoidance and rule driven behaviour at presentation	0.69	0.53–091	0.008
	CET weight control exercise at presentation	0.55	0.40–0.76	0.0002
	CET mood regulation at presentation	0.91	0.68–1.23	NS
	CET lack of exercise enjoyment at presentation	0.97	0.71–1.33	NS
	CET exercise rigidity at presentation	0.68	0.50–0.92	0.013
No eating disorder	CET avoidance and rule driven behaviour at presentation	0.86	0.69–1.08	NS
	CET weight control exercise at presentation	0.88	0.70–1.12	NS
	CET mood regulation at presentation	0.69	0.69–1.73	NS
	CET lack of exercise enjoyment at presentation	1.09	0.81–1.45	NS
	CET exercise rigidity at presentation	0.84	0.65–1.09	NS

*BMI* body mass index, *CET* compulsive exercise test, *CI* confidence interval, *EDE-Q* eating disorders examination-questionnaire

**Table 4:** Prediction of one-year outcome of family-based treatment of 170 adolescents with restrictive eating disorders

Outcome	Predictor	Odds ratio	95% CI	*p*
EDE-Q global score < 2.0	BMI SDS at presentation	0.88	0.63–1.49	NS
	EDE-Q global score at presentation	0.59	0.44–0.81	0.001
	3-month weight gain	1.21	1.04–1.41	0.016
	Weight suppression at follow-up	0.44	0.26–0.74	0.002
	BMI SDS at presentation	0.96	0.62–1.48	NS
	EDE-Q global score at presentation	0.65	0.46–0.90	0.011
	3-month weight gain	1.22	1.04–1.42	0.014
	Weight suppression at follow-up	0.44	0.26–0.74	0.002
	CET avoidance and rule driven behaviour at presentation	0.80	0.55–1.15	NS
	BMI SDS at presentation	1.02	0.66–1.58	NS
	EDE-Q global score at presentation	0.70	0.47–1.02	0.064
	3-month weight gain	1.22	1.04–1.42	0.013
	Weight suppression at follow-up	0.45	0.27–0.77	0.003
	CET weight control exercise at presentation	0.73	0.46–1.16	NS
	BMI SDS at presentation	0.97	0.63–1.50	NS
	EDE-Q global score at presentation	0.58	0.42–0.79	< 0.001
	3-month weight gain	1.21	1.03–1.41	0.017
	Weight suppression at follow-up	0.43	0.26–0.74	0.002
	CET mood regulation at presentation	1.13	0.77–1.66	NS
No eating disorder	BMI SDS at presentation	1.66	1.16–2.38	0.006
	EDE-Q global score at presentation	0.76	0.61–0.95	0.017
	3-month weight gain	1.24	1.09–1.41	< 0.001
	Weight suppression at follow-up	0.73	0.49–1.09	NS
	BMI SDS at presentation	1.66	1.16–2.38	0.006
	EDE-Q global score at presentation	0.77	0.59–0.91	0.043
	3-month weight gain	1.24	1.09–1.41	< 0.001
	Weight suppression at follow-up	0.73	0.49–1.09	NS
	CET avoidance and rule driven behaviour at presentation	0.98	0.74–1.30	NS
	BMI SDS at presentation	1.63	1.13–2.35	0.008
	EDE-Q global score at presentation	0.71	0.53–0.97	0.029
	3-month weight gain	1.24	1.09–1.41	0.001
	Weight suppression at follow-up	0.72	0.48–1.08	NS
	CET weight control exercise at presentation	1.12	0.78–1.60	NS
	BMI SDS at presentation	1.66	1.16–2.39	0.006
	EDE-Q global score at presentation	0.76	0.60–0.96	0.023
	3-month weight gain	1.24	1.09–1.41	< 0.001
	Weight suppression at follow-up	0.73	0.49–1.09	NS
	CET mood regulation at presentation	1.01	0.75–1.35	NS

*BMI* body mass index, *CET* compulsive exercise test, *CI* confidence interval, *EDE-Q* eating disorders examination-questionnaire, *SDS* standard deviation score

## Discussion

The present data confirm the hypothesis that the urge to exercise for weight control and for avoiding low mood decreases with successful family-based interventions for adolescent ED. These two aspects of exercise are closely related to ED cognitions [14, 15] and they appear to decrease in parallel. In contrast, “mood regulation” or exercise for an agreeable experience, which is weakly or not at all related to ED cognitions [14, 15], changed only little during treatment. The scores for “lack of exercise enjoyment” are low and changed only little. This subscale is, however, not related to ED cognitions but rather to low exercise frequency and a general low interest in exercise [14]. The scores for “exercise rigidity” decreased in recovered patients. It is, however, notable that this subscale was not confirmed in a previous factor analysis, is only weakly related to ED cognitions and the items may reflect scheduled organisation of every-day life and not only cognitive inflexibility [14]. An exercise profile typical for recovered adolescents therefore emerges. It is characterized by a substantial reduction of the aspects of exercise closely related to ED cognitions but with retention of the ability to enjoy exercise. In this respect, CET scores of recovered patients resembles those of reference adolescent samples in which the highest scores are for “mood regulation” [12, 27]. It is notable that the change of CET scores is achieved without exercise-specific interventions apart from stopping all exercise at the start of treatment and not reintroducing it until this can be done without provoking ED cognitions.

Contrary to the hypothesis the subscales “weight control exercise” and “avoidance and rule driven behaviour” did not independently predict the one-year outcome of the ED. The outcome “no ED” was not predicted by these CET subscales when they were analysed as single predictors. It is therefore not surprising that they did not add to prediction when forced into the model with the other predictors. The outcome “EDE-Q < 2.0” was predicted by the CET subscales but did not add to prediction in the model with the other predictors. However, “weight control exercise” and “avoidance and rule driven behaviour” are closely related to ED cognitions, which in their own right are important predictors of outcome in this family-based intervention [17]. This suggests that the urge to exercise may not be differentiated from other ED cognitions and decreases in parallel during successful treatment when all ED behaviours are simultaneously and efficiently targeted. The present data does, however, not explain how compulsive exercise and ED cognitions interact. Further studies are needed to elucidate whether ED cognitions drive the urge to exercise or, alternatively, compulsive exercise maintains ED cognitions.

Another important explanation of the lack of independent predictive value of “avoidance and rule driven behaviour” and “weight control exercise” is the intervention against exercise at the start of treatment presently proposed [18]. The advice that all exercise should be stopped is based on the assumption that it is not possible to with certainty distinguish between exercise associated with ED cognitions and exercise for sociable and agreeable effects. The only means to control the ED compulsive exercise and its effect of maintaining ED cognitions would therefore be to stop all exercise. At the start of treatment, the low weight/on-going weight loss and ED ideation are also targets of intervention [18]. For these aspects of the ED a graded response to the interventions would be expected as measured by weight change and change in EDE-Q scores, which then emerge as predictors of outcome. If stopping all exercise is efficiently implemented there will not be a graded response to the intervention. Outcome will not be influenced by the level and intention of exercise at the start of treatment and the exercise-related measures will therefore not add to prediction of outcome.

Continuing exercise at the start of treatment would not only maintain ED cognitions but could also reduce weight gain. Rapid weight gain at the start of treatment predicts a favourable long-term outcome in this and other family-based interventions [17, 28]. Exercise may thus indirectly influence outcome by reducing weight gain, which adds a further argument for stopping exercise at the start of treatment.

A strength of this study is the large number of patients and the short duration of the ED, which had not been treated prior to presentation. Further strengths are that the participants represent almost all patients in the catchment area and are treated at the only ED specialist service available according to a standardised programme [16, 17]. The profile of CET subscale scores and their lack of predictive value for outcome were similar for both outcome measures. Thus, the possible short-comings of self-report (minimization or denial) and interview (judgement error and inter-rater differences) do not appear to influence the conclusions. It is, however, notable that only a minority of the patients fulfilled the weight criterion for AN and that findings may not be generalizable to larger samples of AN only. A short-coming is that all patients were not followed up. We have, however, shown those not followed up in most respects are not different from the sample presently examined [17]. Another problem may be the use of the questionnaire EDE-Q and not the full EDE interview. Although there is considerable agreement between the two measures [29] there may be differences. This is shown by the patients with low EDE-Q scores who were diagnosed with an ED at the follow-up interview. Another problem is that the therapists performing the interviews could be biased since they were not blinded to the treatment, its course and outcome. On the other hand, knowing the patient may help to reveal vague or concealed symptoms of the ED, which otherwise would pass undetected. A further limitation is the lack of a reference population for comparison. These limitations do, however, not preclude the analyses of changes of CET scores in relation to treatment outcome.

The observations have clinical implications. Exercise may be a prominent feature at presentation of the ED and stopping exercise is part of an intervention aiming at disrupting several ED behaviours which could maintain the disease. The approach of stopping *all* exercise simplifies the start of treatment since discussing, planning and monitoring exercise will not be needed. The present study does, however, not address how exercise should be reintroduced. It is unarguable that exercise should not be started before medical stabilization has been achieved. Beyond medical stabilization there may be considerable weight loss to be recovered and remaining strong ED cognitions. Whether a favourable outcome is best promoted by early but carefully monitored introduction of exercise or by abstaining from exercise until partial somatic and cognitive recovery is reached is unclear. Whichever, there may be differences depending on the type and setting of exercise sessions [30, 31]. Considering that the positive and agreeable aspects of exercise remain throughout the course of treatment but can be overshadowed by ED compulsive exercise the reintroduction of exercise need to be addressed in future research.

## Conclusion

Compulsive exercise is a prominent feature of adolescent ED. In the present family-based intervention with a complete stop of all exercise at start of treatment the urge to exercise faded in parallel with other ED related cognitions and behaviours. The ability to exercise for pleasure and enjoyment was, however, retained during treatment.

## Abbreviations

AN: Anorexia nervosa
BMI: Body mass index
CET: Compulsive exercise test
ED: Eating disorder
EDE-Q: Eating disorders examination – questionnaire
EDU: Eating disorders unit
MADRS-S: Montgomery-Åsberg depression rating scale – self report
OSFEDr: Other specified feeding and eating disorders, restrictive subtype
SD: Standard deviation
SDS: Standard deviation score
